# Interactions of HLA-DR and Topoisomerase I Epitope Modulated Genetic Risk for Systemic Sclerosis

**DOI:** 10.1038/s41598-018-37038-z

**Published:** 2019-01-24

**Authors:** Sirilak Kongkaew, Thanyada Rungrotmongkol, Chutintorn Punwong, Hiroshi Noguchi, Fujio Takeuchi, Nawee Kungwan, Peter Wolschann, Supot Hannongbua

**Affiliations:** 1 Program in Biotechnology, Faculty of Science, Chulalongkorn University, Bangkok, 10330 Thailand; 2 The Center of Excellence in Computational Chemistry, Department of Chemistry, Faculty of Science, Chulalongkorn University, Bangkok, 10330 Thailand; 3 Biocatalyst and Environmental Biotechnology Research unit, Department of Biochemistry, Faculty of Science, Chulalongkorn University, Bangkok, 10330 Thailand; 4 Ph.D. Program in Bioinformatics and Computational Biology, Faculty of Science, Chulalongkorn University, Bangkok, 10330 Thailand; 5 Department of Physics, Faculty of Science, Prince of Songkla University, Hat Yai, Songkhla, 90110 Thailand; 6 School of Pharmacy, Nihon Pharmaceutical University, Saitama, 361-0806 Japan; 7 School of Pharmaceutical Sciences, University of Shizuoka, Shizuoka, 422-8526 Japan; 8 Faculty of Health and Nutrition, Tokyo Seiei University, Tokyo, 124-8530 Japan; 9 Department of Chemistry, Faculty of Science, Chiang Mai University, Chiang Mai, 50200 Thailand; 10 Center of Excellence in Materials Science and Technology, Chiang Mai University, Chiang Mai, 50200 Thailand; 11 Department of Pharmaceutical Chemistry, University of Vienna, Vienna, 1090 Austria; 12 Institute of Theoretical Chemistry, University of Vienna, Vienna, 1090 Austria

## Abstract

The association of systemic sclerosis with anti-Topoisomerase 1 antibody (ATASSc) with specific alleles of human leukocyte antigen (HLA)-DR has been observed among various ethnics. The anti-Topoisomerase 1 antibody is a common autoantibody in SSc with diffuse cutaneous scleroderma, which is one of the clinical subtypes of SSc. On the other hand, an immunodominant peptide of topoisomerase 1 (Top1) self-protein (residues 349–368) was reported to have strong association with ATASSc. In this study, molecular dynamics simulation was performed on the complexes of Top1 peptide with various HLA-DR subtypes divided into ATASSc-associated alleles (HLA-DRB1*08:02, HLA-DRB1*11:01 and HLA-DRB1*11:04), suspected allele (HLA-DRB5*01:02), and non-associated allele (HLA-DRB1*01:01). The unique interaction for each system was compared to the others in terms of dynamical behaviors, binding free energies and solvation effects. Our results showed that three HLA-DR/Top1 complexes of ATASSc association mostly exhibited high protein stability and increased binding efficiency without solvent interruption, in contrast to non-association. The suspected case (HLA-DRB5*01:02) binds Top1 as strongly as the ATASSc association case, which implied a highly possible risk for ATASSc development. This finding might support ATASSc development mechanism leading to a guideline for the treatment and avoidance of pathogens like Top1 self-peptide risk for ATASSc.

## Introduction

Systemic sclerosis (SSc) is an autoimmune multisystem disease, clinically characterized by scleroderma, visceral organ fibrosis including lung, kidney, and gastrointestinal tract, microvascular injury, and immune activation with specific autoantibodies^1,2^. The prevalence of SSc is more often in female than in male at a 4:1 ratio, and the highest occurrence appears in the age range around sixty^3–5^. The majority causes of death in SSc patients are fibrosis and pulmonary hypertension^6^. Unfortunately, the etiology of SSc is not well understood, but the possible induction by environmental agents, hormones, genetic factors and irregular immunity is assumed^7,8^. SSc is commonly classified into two clinical subtypes with symptomatic signs; systemic sclerosis with limited cutaneous sclerosis (lcSSc) and systemic sclerosis with diffuse cutaneous sclerosis (dcSSc)^9^. In dcSSc, diffuse scleroderma and the failures in heart, gastrointestinal tract, lung, renal and other internal organs are observed, and the pulmonary hypertension is more common in lcSSc^5,9^. The positive autoantibody testing of anti-topoisomerase I and anti-centromere antibodies are commonly accepted to clinically diagnose for dcSSc and lcSSc subtypes^10–12^, respectively. The anti-Top1 antibody (ATA) directly resisted topoisomerase I (Top1) activity involving an increased collagen transcription^13^. The ATA was frequently observed in dcSSc-patients’ sera who had drastic pulmonary fibrosis and cardiac arrest related to accumulation of collagen deposition in tissues leading to death^14^. Nevertheless, the ATA is more frequent in dcSSc, but is uncommon in lcSSc. Top1 peptides were identified as antigenic determinants associated with SSc positive for ATA (SSc with ATA, ATASSc) using B-cell epitope mapping of ATA response. Interestingly, the twenty-mer sequencing RIANFKIEPPGLFRGRGNHP (349–368) from total 63 Top1 fragments exhibited ATASSc-association by 71% sensitivity and 98% specific binding with ATA^15^. According to the crystalized structure, this sequence is located at an exposed area, where it could be easily recognized by ATA^15,16^.

Although the etiology of SSc is not clarified yet, the contribution of several genetic factors including human leukocyte antigen (HLA)-DR to the pathogenesis of SSc were reported^17–20^. HLA-DR genes correlated with ATA are significantly remarked as the risk factors of ATASSc and dcSSc^18,21–24^. For example, HLA-DRB1*08:02, HLA-DRB1*11:01, HLA-DRB1*11:04 and HLA-DRB1*15:02 associated with ATASSc was observed in Mexican admixed^5^, Caucasian^25^, African-America/Italian-Spanish^26,27^, and Chinese/Thai/Japanese^18,28,29^ patients, respectively. In addition, the strong linkage disequilibrium of HLA-DRB1*15:02 is widely known as HLA-DRB5*01:02 in Thai patients with ATASSc^28^. Because of the very strong linkage disequilibrium, it is difficult to define HLA-DRB5*01:02 as the real susceptible gene. Interestingly, HLA-DRB1*08:02, HLA-DRB1*11:01, HLA-DRB1*11:04 alleles and HLA-DRB5*01:02 allele have the similar amino acids sequence at the hypervariable region of the HLA-DR *β* chain (residues 67–71, FLEDR)^30^. The identical hypervariable motif on HLA-DRB5*01:02 is possibly suggested as susceptibility gene of ATASSc, however the present study specifies a suspect ATASSc. On the other hand, HLA-DRB1*01:01 does not relate with the ATA, but it is instead specific to the ACA associated with SSc among Caucasian and Japanese ethnics^25,29^.

**Figure 1:**
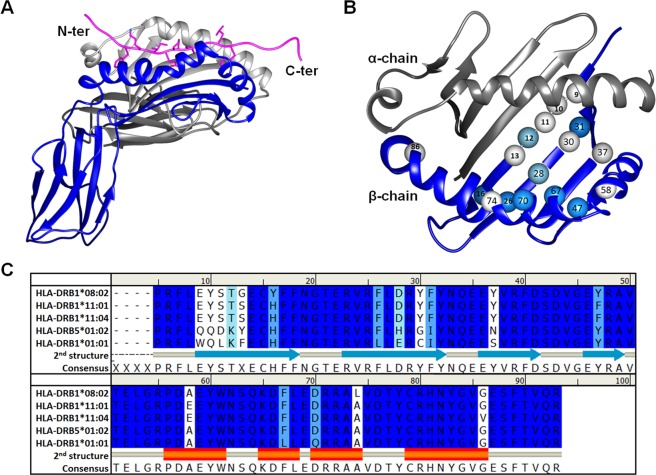
Basic structure of class II HLA. (**A**) HLA-DR is constructed of *α-* (gray) and *β*- (blue) chains to restrict antigenic peptide (magenta ribbon). (**B**) Polymorphic residues of HLA-DR *β*-chain are marked as spheres on the top view of binding cleft. (**C**) Sequence alignment of HLA-DR *β*-chain, whereas the residues of HLA-DR *α-*chain are conserved (data not shown).

In principle, HLA-DR alleles are members of HLA class II out of total three classes connected with immunological procedures. The HLA class II molecules play a major role in antigenic presentation expressed on the cell surface to CD4+ T helper (T_h_) cell^31^. An antigen is recognized by T-cell signaling to secrete specific cytokines from other immune cells^32^. As is presented by molecular structure, HLA-DR is a heterodimer consisting of *α* (DRA) and *β* (DRB) chains (Fig. 1A). Two chains are assembled with non-covalent interaction to form an antigenic binding cleft containing eight *β*-sheets between two anti-parallel *α*-helices (Fig. 1B). Alpha chain contains conserved residues for many HLA-DR molecules^31^. Diversity of amino acids is found on *β*-chains, specifically at the binding cleft region which represents various HLA-DR types as shown in Fig. 1C. The binding cleft of HLA class II can accommodate a diverse antigenic peptide in the length of 12 to 20 amino acids with a flattened shape. The residues at the middle range of the peptide; p1, p4, p6 and p9 are mounted on the binding cleft by inducing a change in shape and size to be well fitted in each sub-pocket^33^. In healthy cell, the immune system protects the body by detecting “foreign” –peptides such as viral or bacterial fragments, while self-tissues are ignored. In case of autoimmune diseases, the self-tissues are misrecognized as “foreign” –peptides by immunity such as in the case of SSc disease^34^. Recently, HLA-DR alleles and Top1 were reported to be susceptible to ATASSc and led to the description of binding interactions in ATASSc pathogenesis^8^.

This study challengingly attempted to identify and characterize the relation of self-antigenic (RIANFKIEPPGLFRGRGNHP) peptide from Top1 protein in selective binding with HLA-DRB1*08:02, HLA-DRB1*11:01, HLA-DRB1*11:04, HLA-DRB5*01:02 or HLA-DRB1*01:01 which could individually contribute to SSc disease risk at different levels. Based on the HLA function and genetic markers, the interaction of HLA-DR/Top1 was evidently investigated by molecular dynamics (MD) simulations. Indeed, molecular understanding of the binding recognition between the motif of self-peptide and HLA-DR alleles is useful for the avoidance of infectious agents carrying epitope such as a self-antigen (Top1) that causes SSc disease. Furthermore, this could be used for drug design against SSc by HLA/self-peptide inhibition.

## Results

### Dynamics behavior of HLA-DR complexed to Top1 peptide

**Figure 2:**
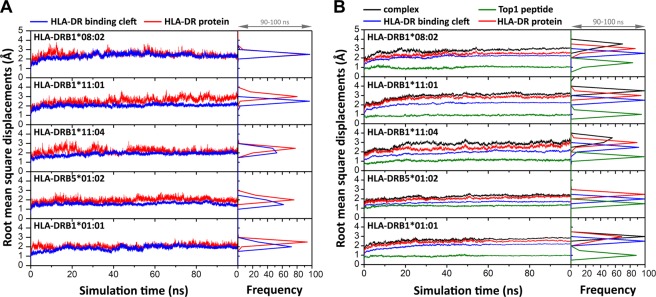
RMSD on time series of HLA-DRs. (**A**) A variety of HLA-DRs without and (**B**) with Top1 binding are separately plotted for RMSD of the backbone atoms for HLA-DR/Top1 complex (black), HLA-DR protein (red), binding cleft (blue) and nonameric core sequence of Top1 peptide (green). Distributions of RMSD distance during productive selection of 90–100 ns for HLA free form and complex form are collected as the relative frequency.

Root-mean-square displacement (RMSD) calculations were performed to monitor the conformational stability in the overall MD simulation, as shown in Fig. 2. RMSD along simulated time was evaluated from the geometrical coordinates of protein backbone (N-C*α*-C-O) with respect to those of the initial structure. For HLA-DRs without Top1, RMSDs for the binding cleft and the whole protein were plotted in Fig. 2A. In the first 2-ns, RMSD value rapidly reaches up to 2 Å for HLA-DR binding cleft and whole protein in every system, and consequently establishes dynamics equilibrium. For the complex form (Fig. 2B), HLA-DR/Top1 complex, HLA-DR, binding cleft and nonameric core sequence of Top1 peptide are separately considered by averaging 10 trajectories. Similar curves are observed for the complex and the HLA-DR protein (black and red in Fig. 2B), indicating that the principle dynamics in the system largely depend on HLA-DR part. The systems of HLA-DR/Top1 complex show the RMSD values of ~2–3 Å with ~0.3 Å of fluctuation. This is due to a large size of HLA-DR molecules composing two major domains, which are an antigenic binding cleft and the cleft-distal domain interacting with a CD4+ T-cell receptor. Structural flexibilities result from peptide flanking regions and cleft-distal domain while the binding cleft maintains the movement within ~0.2 Å. From the RMSDs of binding cleft (blue) and nonameric core sequence of Top1 (green), HLA-DR/Top1 complex and HLA-DR free form are likely stable after 50 ns and 55 ns, respectively. Besides, the distance between the centers of gravity of 9-mer core Top1 peptide and HLA binding cleft is also monitored. It can be seen from the Supplemental Fig. S1 that the distance of all complexes has no significant change after 55 ns. From RMSD and the distance plots, the equilibrium phase is appropriately considered after the simulation reaches 10-ns for HLA-DR/Top1 complex and HLA-DR free form. Several sections during equilibrium phase of HLA-DR and HLA-DR/Top1 systems are rechecked by the RMSD frequency. The high frequency of distribution displacement within 1 Å is properly examined to resolve the orientation for productive sampling, especially for HLA-DR binding cleft and Top1 peptide. The conformational selections within equilibrium production are investigated for binding interaction of HLA-DR/Top1 complexes and dynamical behavior of free HLA-DRs.

**Figure 3:**
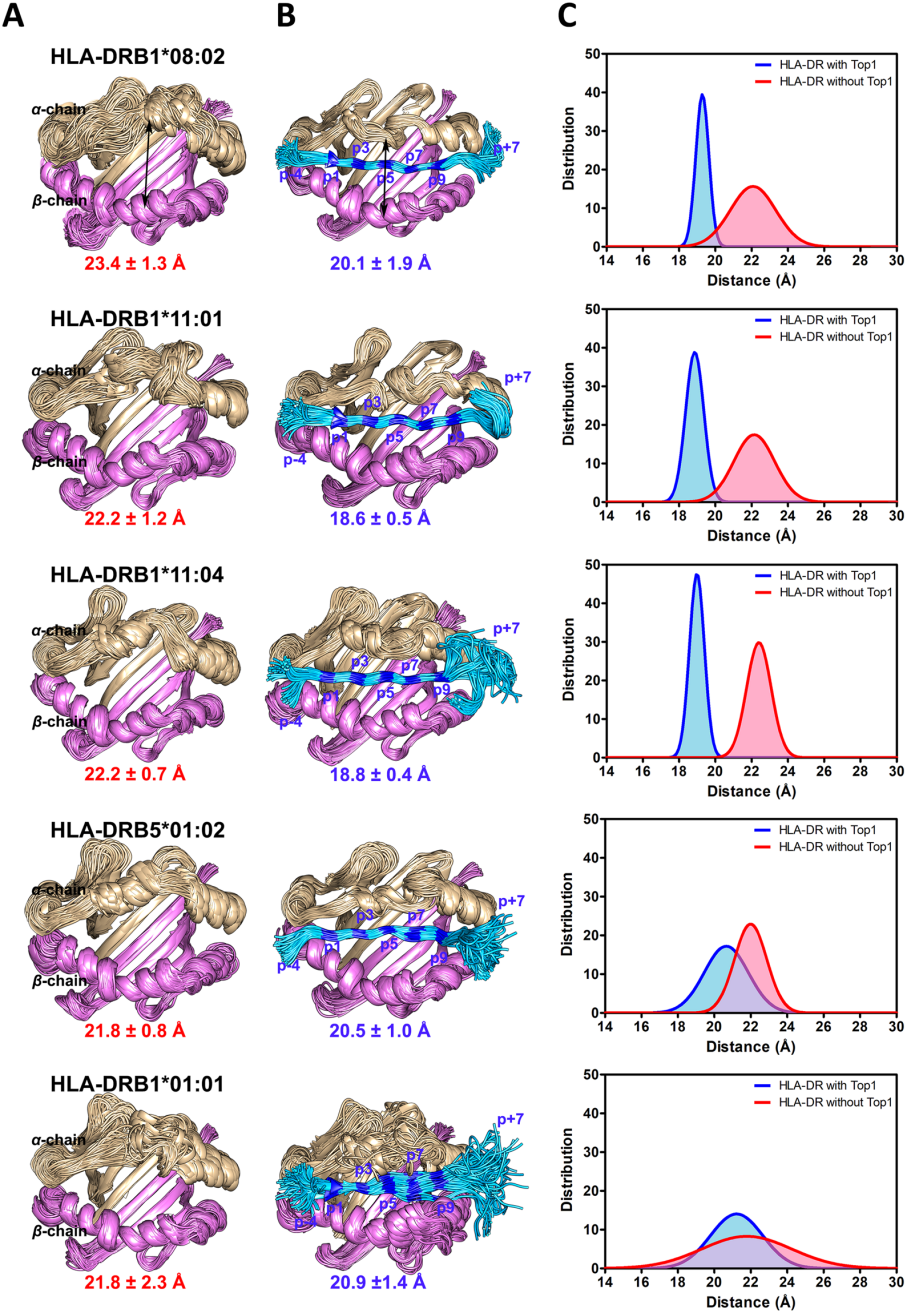
Comparison of HLA-DR binding clefts without and with Top1 peptide (cyan-blue multicolor) in columns (**A** and **B**), respectively. Column (**C**) represents distribution of the distance between *α*-chain and *β*-chain of the binding clefts. The distance distribution of HLA-DR in free form is shown as a red peak and that for the binding peptide is shown as a blue peak.

To consider flexibility of the binding cleft in cases of Top1 bound and unbound, the set of 250 snapshots is superimposed within their simulated systems. In Fig. 3A,B, the binding cleft consists of the *α*-chain (residues 5–80 shown in tan ribbon) and the *β*-chain (residues 5–93 in pink ribbon), while the cleft-distal domains are not displayed. Each chain is constructed by four antiparallel sheets and one long helix. Empty HLA-DR clefts are depicted in the first column (Fig. 3A), and the next column represents individual HLA-DR of the same row binding with Top1 (Fig. 3B). For the molecular set of HLA-DR types, the superimposition of empty clefts exhibits open shape and high flexibility, especially at the helix and turn/coil parts on *α*-chain. The binding cleft manifests lower flexibility at the turn and coil conformations on *α*-chain when binding with Top1 peptide due to an induced-fit mechanism. For HLA-DR types with ATASSc association, the core length (p1–p9) of Top1 peptide orderly lies on the binding cleft, while two ends extend out of the cleft with a free motion. Unsurprisingly, Top1 peptide shows the highest flexibility in HLA-DRB1*01:01 complex (non-associated case).

To support this finding, the distance between the two centers of gravity (CG) of *α*-helixes are measured to represent a cleft size of HLA-DR with and without Top1 peptide bound. Overview of average distance is estimated to be in a range of 21.8–23.4 Å without peptide and becomes somewhat shorten to 18.6–20.9 Å with peptide binding. The effect of induced-fit with Top1 peptide leads to two *α*-helixes approaching each other only found in HLA-DRs associated and suspected ATASSc. By a comparison within identical HLA-DR molecules, the difference in the average distances of the binding cleft with and without Top1 are indicated in the parentheses as follows: HLA-DRB1*08:02 (−3.3 Å), HLA-DRB1*11:01 (−3.5 Å), HLA-DRB1*11:04 (−3.4 Å), HLA-DRB5*01:02 (−1.3 Å) and HLA-DRB1*01:01 (−0.8 Å), where the negative values are referred to smaller distances of binding cleft upon Top1 binding, respectively. In Fig. 3C, the profile of normal distribution of distance is quite similar for almost all HLA-DRs that are a broad peak for free protein and a narrow peak shifting to the shorter distance for the Top1-bound cases. According to the high protein flexibility, this could explain why the peak is spread out in the free form of HLA-DRs. The binding clefts become more stable by complexation with Top1, which is illustrated by a narrow peak of the distance distribution. More oscillatory configuration and the peak of distance distribution shifting to the extended distance suggest the less-fit Top1 in non-ATASSc binding cleft for HLA-DRB1*01:01.

### Binding free energy calculations

MM-PB(GB)SA and QM/MM-GBSA binding free energy calculations were applied on the HLA-DR/Top1 complexes to quantify the antigenic binding strength in diverse antigen presenting types using 250 equilibrated frames for each replica. Binding free energy (∆*G*_*bind*_) based on MM-PB(GB)SA is contributed by enthalpy and entropic terms at a constant temperature (-T∆*S*). The enthalpy term contains molecular mechanics energy (Δ*E*_*MM*_), and the solvation term (∆*G*_*sol*_). The latter one comprises of polar and nonpolar solvation energies. The energy components are enumerated in Table 1. In gas phase, the electrostatic (∆*E*_*ele*_) energy mainly contributes for the binding interaction between HLA-DR and Top1 peptide, which is over four times stronger than the van der Waals (∆*E*_*vdW*_) attraction. The most stable complex is HLA-DRB5*01:02/Top1 (the suspect system with MM energy of −906.9 kcal/mol), while three HLA-DRs with ATASSc-association systems show such ∆*E*_*MM*_ values ranging from −733.4 to −715.6 kcal/mol. The non-associated ATASSc protein, HLA-DRB1*01:01, has the lowest protein-protein interaction according to the ∆*E*_*MM*_ term of −630.7 kcal/mol. Cooperation of enthalpy and entropy invoked from binding free energy clearly exposes that Top1 peptide had strong binding with ATASSc-associated HLA-DRB1*08:02 (−52.7 kcal/mol), HLA-DRB1*11:01 (−47.0 kcal/mol), HLA-DRB1*11:04 (−47.8 kcal/mol), ATASSc-suspect HLA-DRB5*01:02 (−51.2 kcal/mol) and rather weak binding with ATASSc-unassociated HLA-DRB1*01:01 (−40.9 kcal/mol), according to MM-PBSA approach. MM-GBSA binding free energies reveal similar tendency as stated in Table 1. Surprisingly for ATASSc-suspect, the HLA-DRB5*01:02/Top1 has the tightest binding interaction among HLA-DRs studied here. However, the self-antigen is only slightly bound to non-associated with ATASSc type, HLA-DRB1*01:01, as hypothesized. From the obtained results, it can be implied that not only HLA-DRB1*15:02 is the known gene expression in Thai-SSc patients but the strong linkage disequilibrium of HLA-DRB5*01:02 also is related to SSc disease. Works on the clinical data to confirm the relation between the genes linkage disequilibrium and the severity of the SSc disease are in progress.

**Table 1:** The relative binding free energy and energy components (kcal/mol) for the five HLA-DR/Top1 complexes predicted by the MM-PB(GB)SA and QM/MM-GBSA methods.

Energy Component	HLA-DRB1*08:02^a^	HLA-DRB1*11:01^a^	HLA-DRB1*11:04^a^	HLA-DRB5*01:02^b^	HLA-DRB1*01:01^c^
**Gas term**					
∆*E*_*vdW*_	−143.5 ± 2.3	−134.2 ± 1.6	−139.2 ± 3.0	−137.4 ± 1.1	−132.4 ± 2.0
∆*E*_*ele*_	−584.7 ± 25.4	−599.1 ± 9.0	−576.4 ± 25.9	−769.5 ± 14.3	−498.3 ± 22.9
∆*E*_*MM*_	−728.2 ± 25.5	−733.4 ± 8.1	−715.6 ± 27.3	−906.9 ± 15.2	−630.7 ± 23.6
*∆E*_*QM*_	−22.5 ± 2.5	−33.9 ± 1.0	−36.7 ± 0.8	−47.4 ± 4.0	−24.6 ± 1.7
−T∆*S*	65.8 ± 1.7	66.2 ± 1.2	67.9 ± 1.2	69.3 ± 1.8	73.6 ± 2.3
**Solvation term**					
∆*G*_*sol(PBSA)*_	609.7 ± 25.0	620.1 ± 7.1	600.0 ± 24.6	786.3 ± 13.1	516.2 ± 22.6
∆*G*_*sol(GBSA)*_	608.8 ± 23.8	613.3 ± 7.3	596.6 ± 25.4	780.0 ± 13.1	517.0 ± 21.5
∆*G*_*sol(QM-GBSA)*_	629.2 ± 23.7	652.3 ± 8.1	623.8 ± 24.7	819.2 ± 12.2	575.8 ± 23.0
**Binding free energy**					
∆*G*_*bind(MM/PBSA)*_	−52.7 ± 3.3	−47.0 ± 1.2	−47.8 ± 3.5	−51.2 ± 2.7	−40.9 ± 2.1
∆*G*_*bind(MM/GBSA)*_	−53.6 ± 1.4	−53.4 ± 0.6	−51.1 ± 1.7	−57.6 ± 2.7	−40.1 ± 1.2
∆*G*_*bind(QM/MM-GBSA)*_	−33.4 ± 1.4	−36.5 ± 0.6	−33.2 ± 1.8	−37.5 ± 2.3	−21.2 ± 1.7
**Relative binding free energy**					
∆∆*G*_*bind(MM/PBSA)*_	−11.8	−6.1	−6.9	−10.3	0
∆∆*G*_*bind(MM/GBSA)*_	−13.5	−13.3	−11.0	−17.5	0
∆∆*G*_*bind(QM/MM-GBSA)*_	−12.2	−15.3	−12.0	−16.3	0

The complexes of Top1 self-peptide and HLA-DR molecules for ^a^association, ^b^suspect, and ^c^non-association with ATASSc. Mean ± std.err. of mean is estimated from 10 independent simulations. The MM-PB(GB)SA and QM/MM-GBSA calculations are applied on 250 snapshots, whereas NMODE are performed into entropic terms on 10 snapshots. The ∆∆*G*_*bind*_ term represents the relative binding free energy which are calculated with regard to the non-association ∆*G*_*bind*_ result.

To strengthen the validity of the calculated binding energies for HLA-DR/Top1 complexes, the higher-level computation was applied using QM/MM-GBSA method. In QM region, the nonameric core sequence of Top1 peptide was treated with SCC-DFTB implementation. The ∆*G*_*bind(QM/MM-GBSA)*_ confirmed that the major HLA-DR binding cleft interacts within the nine core residues (p1 to p9) of Top1 peptide. Nonameric residues, p1 to p9, have conserved interactions with the HLA-DR cleft of associated (in a range of −33.2 to −36.5 kcal/mol) and suspect ATASSc (−37.5 kcal/mol) types; in contrast, they are barely attracted on the non-associated allele at −21.2 kcal/mol. The relative binding free energies (∆∆*G*_*bind*_) are calculated with regard to HLA-DRB1*01:01 as the lowest value of 0 kcal/mol, as shown in Table 1. Evidently, the other HLA-DR systems are justified into the same group with close relative binding energy. Explanation for HLA-DR/Top1 binding is going to be more enlightened by per-residue energy decomposition and hydrogen bonding across protein-protein interface.

### Per-residue energy decomposition

**Figure 4:**
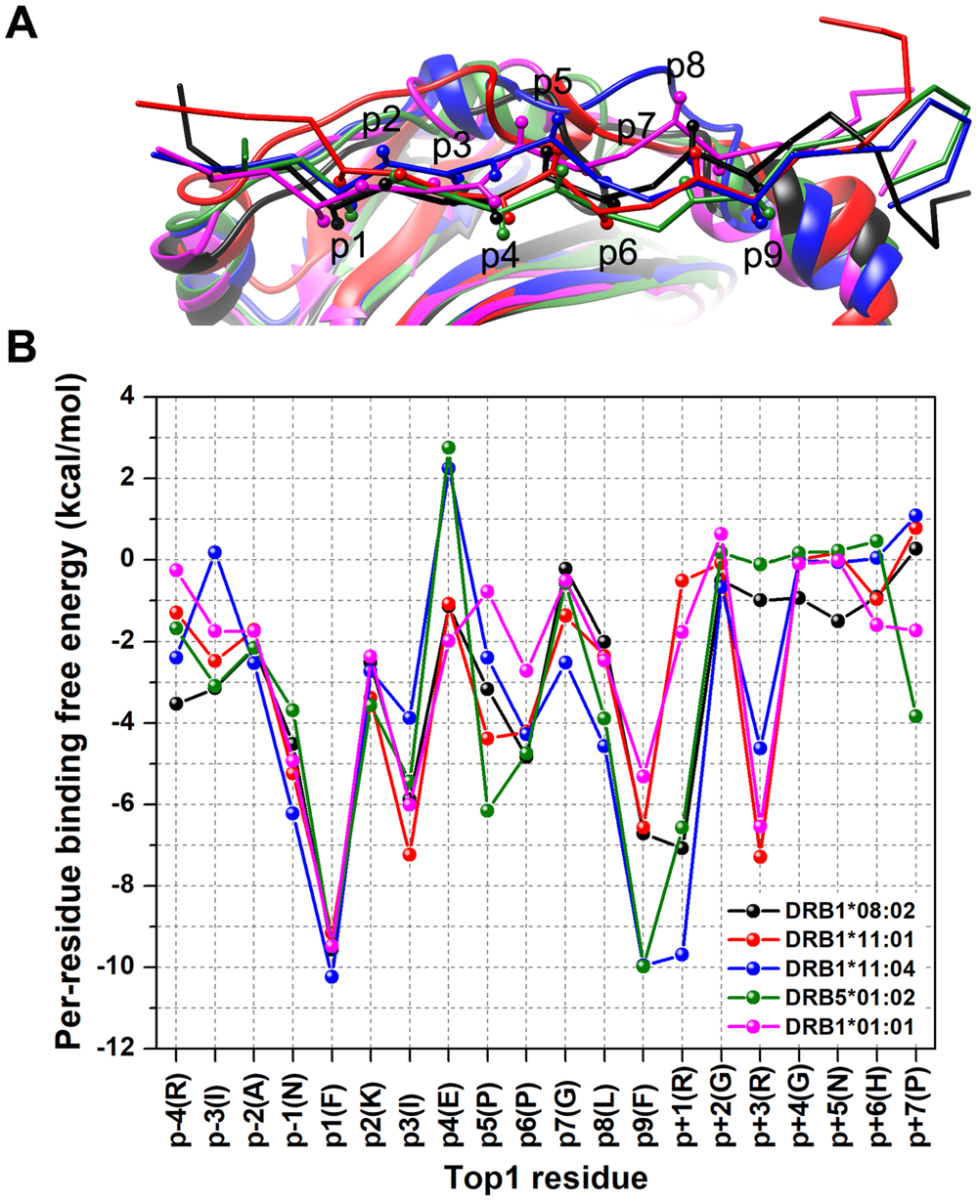
Top1 peptide binding with various HLA-DR molecules. (**A**) A superimposition of representative Top1 in five MD systems showing a side view of HLA-DR binding cleft. The Top1 binding to HLA-DRB1*08:02 is shown in black, HLA-DRB1*11:01 in red, HLA-DRB1*11:04 in blue, HLA-DRB5*01:02 in green and HLA-DRB1*01:01 in magenta. The Top1 peptide is depicted by the backbone chain and CB atoms of the side chains as a stick and a ball, respectively. (**B**) Per-residue binding free energy of Top1 peptide for all five HLA-DRs.

According to the structural database, the peptide motifs complexed to HLA class II were generally believed to have the same burial pattern with the key residue contacts at p1, p4, p6 and p9^33^. To determine this point, the structural superimposition over all studied systems taken from the last MD snapshot was carried out. By a comparison in the side chain directions, *i.e*. carbon beta (CB) atom in Fig. 4A, five HLA-DR molecules are predicted for possible key anchors of Top1 peptide. The CB atoms of p1, p4, p6 and p9 are likely to face the binding cleft.

The relative binding affinity of Top1 in various HLA-DR types is examined by per-residue energy decomposition using implicit solvent model. The total binding free energy excluding entropic contribution is plotted per-residue in Fig. 4B to illustrate the Top1 binding pattern. The binding free energy of individual amino acids within the core 9-mer is compared at identical position among all five systems. Energetic plot per residue shows similar tendency of Top1 binding interaction on diverse HLA-DR molecules (Fig. 4B). Phenylalanine at the p1 and p9 positions clearly shows a strong interaction with HLA-DR with the binding free energy of <−9 and −6 kcal/mol, respectively. On the other hand, the p9 phenylalanine exhibits a weaker binding (~−5 kcal/mol) to HLA-DRB1*01:01 insusceptible to ATASSc than the other antigenic presenter molecules. Decreasing interaction of non-association complex is shown for p5, p6 and p9 (Fig. 4B). The residues concerning HLA-DRs’ sub-pockets confer a significant effect to Top1 interaction; therefore, the residues within 5 Å of core 9-mer bound region of Top1 are focused in which the residues with total decomposition energy ≤−1 kcal/mol are compared in Supplemental Fig. S2. With energy subdivision, the total energy is decomposed into two terms, polar (∆*E*_*ele*_ + ∆*G*_*polar*_) and nonpolar (∆*E*_*vdW*_ + ∆*G*_*nonpolar*_) contributions. Overall Top1’s residues are mainly stabilized by HLA-DR protein through nonpolar interactions.

**Figure 5:**
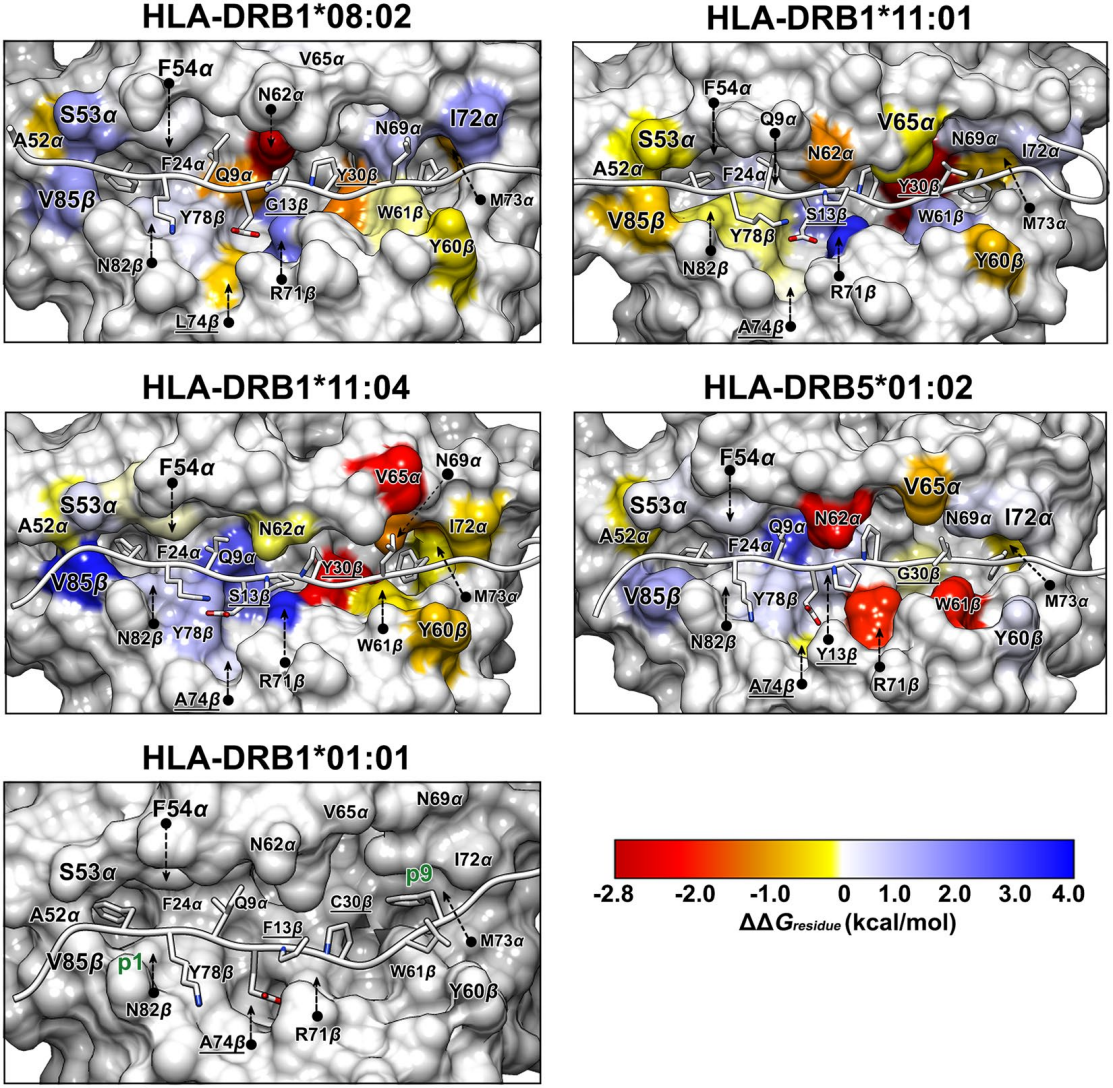
Energetic fingerprints of difference (ΔΔ*G*_*residue*_) in per-residue free energy decomposition values of HLA-DR/Top1 complexes compared to non-associated with ATASSc HLA-DRB1*01:01. Energy contribution of HLA-DR residues for Top1 binding is illustrated by surface coloring. By comparing to per-residue decomposition energy of HLA-DRB1*01:01, the stronger binding is colored from yellow to red gradient and in contrast the weaker binding is shown in blue gradient.

In Fig. 5, energetic fingerprints of difference (ΔΔ*G*_*residue*_) are represented as color-coded surface models of various HLA-DR binding clefts interacting with Top1 in comparison to the HLA-DRB1*01:01/Top1 $({\rm{\Delta }}{G}_{residue}^{HLA-DR}-{\rm{\Delta }}{G}_{residue}^{HLA-DRB{1}^{\ast }01:01})$. The HLA-DRs equally interact to p1 by hydrophobic residue cluster comprising of F24_*α*_, A52_*α*_ and F54_*α*_. A hydrophilic S53_*α*_ on *α*-chain and V85_*β*_ on *β*-chain of HLA-DRB1*01:01 exert tighter binding than HLA-DRB1*08:02, HLA-DRB1*11:04 and HLA-DRB5*01:02 which exhibit adversative representation for HLA-DRB1*11:01 surface. Pi-pi stacking and pi-alkyl interactions are main stabilizations for the p1(F) side chain. The p2(K) and p3(I) are maintained on the cleft supported by N82_*β*_ and pi-alkyl interaction with F54_*α*_, respectively. For some HLA-DR cases, the glutamate group of p4(E) interacted competitively between a positive charge attraction of R71_*β*_ side chain and a negative charge repulsion of neighboring D70_*β*_. Repulsive force on p4(E) is observed in HLA-DRB1*11:04 associated and HLA-DRB5*01:02 suspected ATASSc (Fig. 4B), whereas in the other cases the electrostatic repulsion is lessen owing to Q9_*α*_ stabilization (Supplementary Fig. S2). The middle cleft region surrounding p4(E) for HLA-DRB1*08:02, HLA-DRB1*11:01, HLA-DRB1*11:04 and HLA-DRB5*01:02 have lower or a little bit better binding interaction than HLA-DRB1*01:01 as respectively presented in blue or light yellow colored surface in Fig. 5. Besides, auxiliary interactions come from the adjacent residues of p4(E) like p3(I) and p5(P). The HLA-DRB1*01:01 carries Q70_*β*_ that can accommodate p4(E) without electrostatic repulsion. The pyrrolidine side chain, a nonpolar group, of p6(P) on Top1 is mostly buried within the pocket with a total free energy in the range from −5 to −4 kcal/mol, while only the HLA-DRB1*01:01 case shows the free energy around −2.7 kcal/mol (Fig. 4B). Energetic contributions for p6(P) are partially participated by the residues 62_*α*_, 13_*β*_ and 30_*β*_ depending on the protein type. One of polymorphic residues is G13_*β*_ for HLA-DRB1*08:02, S13_*β*_ for HLA-DRB1*11:01 and HLA-DRB1*11:04, Y13_*β*_ for HLA-DRB5*01:02, and F13_*β*_ for HLA-DRB1*01:01. Sharing contribution of 30_*β*_ interaction between p6 and p9 is verified on the protein surface (Fig. 5). Residue 30 partially constructing the p9-pocket plays an important role in efficient p9 holding of each protein. This pocket consists of nonpolar or aromatic residues and could contribute to the p9(F) anchor interaction with strong binding level, reducing in the non-association case (Fig. 4B). By exerting comparable nonpolar/aromatic effects, Y30_*β*_ is presented for three HLA-DRs associated ATASSc, which differed to the small size G30_*β*_ for ATASSc suspect and the polar C30_*β*_ for non-ATASSc association. This leads to elude p9(F)-interaction in HLA-DRB1*01:01. The remaining residues of Top1 (p5, p7 and p8) within HLA-DRB1*01:01 complex is estimated to have small binding interaction with similar trend of the other four HLA-DRs, as revealed in Fig. 4B. Overall energetic fingerprints normalized with HLA-DRB1*01:01/Top1 are displayed in yellow to red tone at the pocket surrounding p9 of four HLA-DRs, especially at M73_*α*_ deep inside the pocket. Although the ATASSc-suspect system, HLA-DRB5*01:02/Top1 has quite unlike amino acids from ATASSc association type, it nevertheless presents a resembling energy contribution to them.

### Hydrogen bonds across protein-protein interface

**Figure 6:**
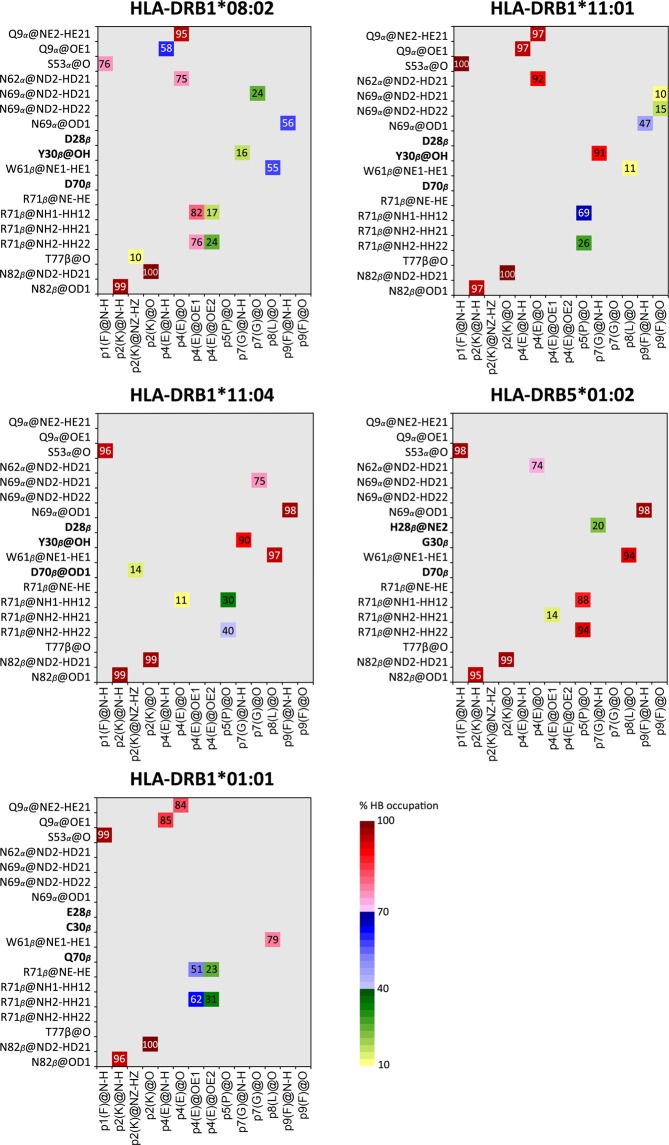
Hydrogen bond (HB) interactions of HLA-DR/Top1 complexes. HBs between HLA-DR (vertical axis) and Top1 (horizontal axis) residues are presented by grid map. Atom types are specified by tleap AMBER conversion. HB strength is defined by label and color in the grid cells, while salt bridge (SB) interaction is framed by a black border.

A significant stabilizing contribution for HLA-DR/Top1 recognition occurrences is by salt bridge (SB) and hydrogen bond (HB) interactions at the protein-protein interface as seen in the gas phase energetic components for total binding free energy (Table 1) that the electrostatic interaction is highly contributed for HLA-DR/Top1 binding. Stabilization by hydrogen bonding interactions is determined by the percentage of occupation based on the criteria for the distance of hydrogen bond donor and acceptor atoms (HD…A) of ≤3.5 Å and the D-H…A angle of >120°. HB occupations between Top1 and sub-pocket residues are exhibited by grid cells in Fig. 6. The hydrogen bond strengths are divided into 3 levels: low (10–39%), moderate (40–69%), and strong (70–100%) interactions represented by the gradient of greenish, bluish and reddish grid cells, respectively. The nonameric core peptide consisting of –FKIEPPGLF– is buried in the antigenic binding cleft, while the rest of the residues on –NH_3_^+^ and –COO^−^ termini stay away from the cleft.

A similar HB pattern across protein-protein interface is found in all complexes by a strong interaction between the backbone atoms of p1(F) to S53_*α*_ and p2(K) to the N82_*β*_ amide group. In HLA-DRB1*08:02/Top1 complex, the acidic side chain of p4(E) is repulsed by the negative charge from D70_*β*_; meanwhile, a salt bridge is found with the R71_*β*_ guanidinium group. The charge-charge repulsion of D70_*β*_-p4(E) leads to a decreased HLA-DR recognition according to the per-residue energy decomposition (Fig. 4). However, the p4(E) backbone atoms form strong hydrogen bonds with the Q9_*α*_ and/or N62_*α*_ amide groups in the binding cleft which compensates Coulomb repulsion for p4(E) in case of HLA-DRB1*08:02, HLA-DRB1*11:01 and HLA-DRB1*01:01. To assist conservative flanking by neighboring p4(E), p5(P) are stabilized by R71_*β*_ for HLA-DRB1*11:01, HLA-DRB1*11:04 and HLA-DRB5*01:02. Since the amino acids at 28_*β*_, 30_*β*_ and 70_*β*_ in the focused systems are distinct, the detected hydrogen bond strengths are different. The Y30_*β*_ hydroxyl group in HLA-DRs with ATASSc-association strongly stabilizes p7(G), because it has a HB acceptor group and a longer side chain than the other two residues: G30_*β*_ for HLA-DRB5*01:02 and C30_*β*_ for HLA-DRB1*01:01, whose side chains are unable to form a HB. p7(G) is found to be weakly stabilized by H28_*β*_ instead for the suspect system. Hydrogen bond networks on the N69_*α*_ amide group and W61_*β*_ are linked to the backbone of p7(G), p8(L) and p9(F). To examine the overall hydrogen bond interactions for different HLA-DR in recognizing Top1 peptide, the hydrogen bonds formed across the protein-protein interface in individual systems are counted and showed strong occupation ranging from 71 to 100%, as illustrated in Fig. 6 with the numbers of reddish cells of 7 (HLA-DRB1*08:02), 7 (HLA-DRB1*11:01), 7 (HLA-DRB1*11:04), 8 (HLA-DRB5*01:02) and 6 (HLA-DRB1*01:01). Interestingly, the hydrogen bond interaction between the peptide and N69_*α*_ is absent in the case of the insusceptible HLA-DRB1*01:01, making the overall number of hydrogen bond pairs lowest among all systems. However, even though majority of Top1 from p1 to p9 are nonpolar residues, hydrogen bond and salt bridge interactions still appeared as additionally supporting interactions. Polar energy contribution is in agreement with strong hydrogen bond network and rather appeared in protein complexes of systematic ATASSc-suspect. Expansion beyond the nonameric core peptide at two sides mostly exhibits temporary occupation of hydrogen bonds with unexpectedly strong occupancy in a few bonds (Supplementary Fig. S3).

### Water solvation effect for self-peptide binding

**Figure 7:**
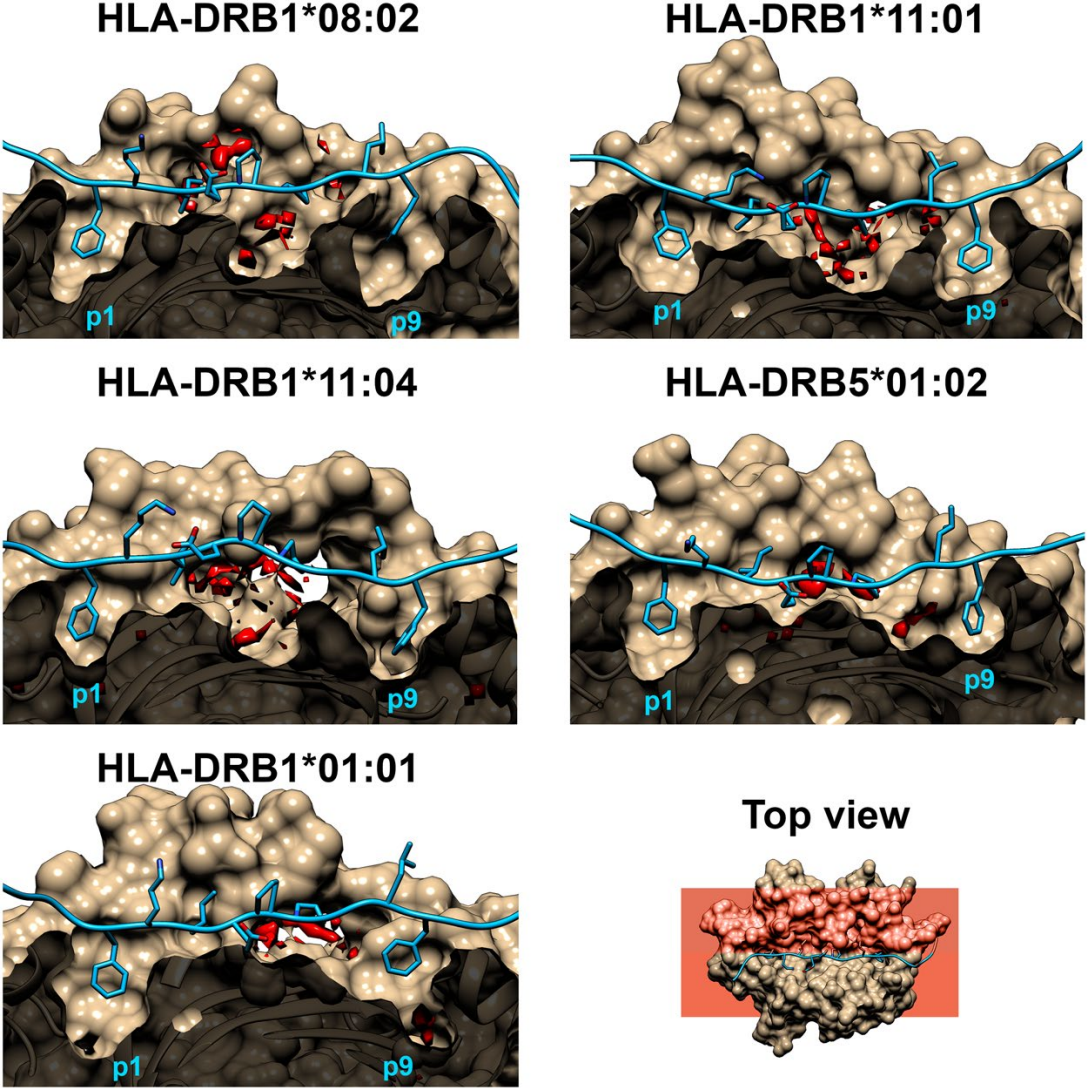
3D distribution function of water oxygen atoms in the cross-sectional plane of HLA-DR/Top1 with g(r) >3. HLA-DRs are sliced off at the binding cleft by a red plane perpendicular to a top view. A zoom-in of a side view represented by a tan surface is the sites specifically supporting p1 and p9 anchors. The core-nonameric residues of Top1 peptide embedded in the binding cleft is represented by a blue ribbon for the backbone and by a stick model for the side chains.

Distribution of water accessibility at HLA-DR/Top1 interface was examined by 3D-RISM-KH molecular theory of solvation^35,36^ with TIP3P model using the rism3d.snglpnt module. Based on like-dissolve-like principle, a hydrophilic protein surface should find a higher amount of water than hydrophobic zone. As already pointed out before, the p1(F), p3(I)/p4(E), p6(P) and p9(F) of Top1 peptide are anchored on HLA-DR binding cleft. Water molecules accessibly inserted between these putative anchor residues and their surrounding residues of HLA in the binding cleft likely affect protein-protein binding strength. To predict the hydration sites around the antigenic binding, the stripped snapshots of peptide-protein complexes are inspected by 3D-RISM model. In 3D maps, the oxygen atoms of water molecules (in red flakes) are represented by 3D isosurfaces with a value >3 of g(r) level (Fig. 7), while hydrogen atoms are not shown. Water occupation is illustrated within HLA-DR sub-pockets around the nonameric core of Top1 peptide. Binding surface of HLAs class II is widely known to consist of flat and shallow sub-pockets, making it easy to be accessible by water molecules. Among all sub-pockets for Top1 binding in Fig. 7, there are two rather deep sub-pockets supporting p1(F) and p9(F) anchors. Their phenyl ring is perfectly buried in the sub-pockets without water molecules accessibility. In contrast for non-associated ATASSc, HLA-DRB1*01:01 complex has a high possibility of accessible waters inside the p9-pocket to compete with the peptide-protein interaction that agrees well with a lesser binding affinity of p9 in Fig. 4B. This site of the ATASSc-insusceptible protein is constructed of specific hydrophilic residues C30_*β*_ and S37_*β*_, which differed from the other HLA-DR molecules. Even though the residue 37 on the *β*-chain of HLA-DRB5*01:02 is asparagine, its side chain points away from p9 making the hydrophobic pocket. For the three ATASSc-susceptible proteins, the –OH tail of Y37_*β*_ also aligns away from p9(F).

## Discussion

Complexation of HLA/peptide is a prerequisite for T-cell recognition leading to an immune response against antigens. In this study, the five different HLA-DRs with a binding of the self-antigen of Top1 protein (RIANFKIEPPGLFRGRGNHP) for ATASSc were characterized. From the obtained dynamical behaviors and binding interactions, HLA-DRB1*01:01 was likely unspecific to Top1 self-peptide which was a candidate for auto-antigenic ATASSc. In comparison between the binding clefts of HLA/peptide complexes and identical HLA free forms, the binding of Top1 peptide has led to a more stable protein similar to the ankylosing spondylitis-associated HLAs with bound peptide^37^. A higher flexibility of the whole complex and the opening of the binding cleft for HLA-DRB1*01:01/Top1 (insusceptible ATASSc), while this flexible magnitude was presented in quite smaller values for the other complexes. The determined Top1 rigidity on various binding clefts suggested that HLA (-DRB1*08:02, -DRB1*11:01 and -DRB1*11:04) associated and (-DRB5*01:02) suspect ATASSc could keep a tighter binding to the core self-peptide than that of the non-associated one. The estimation of the total binding free energy of the peptide and HLA interactions based on either MM-PB(GB)SA for the whole Top1 (20 amino acids) or QM/MM-GBSA for the nonameric core peptide treated by QM method showed a better binding strength for susceptible ATASSc with HLA-DRB1*08:02, HLA-DRB1*11:01 and HLA-DRB1*11:04 than insusceptible ATASSc with HLA-DRB1*01:01. The suspect ATASSc with HLA-DRB5*01:02 presented binding strength as strong as susceptible ATASSc group. The question how different interactions could influence the binding of HLA-DR/Top1 complexes was investigated. We found that minimal side chain with beta carbon atoms of p1, p4, p6 and p9 properly faced at the HLA-DR interface, while the rest turned to the T-cell receptor recognition in accord with the frequent finding for HLA class II binding. Analysis of energetic per-residue decomposition of Top1 indeed identified the important anchors for p1, p3, p6 and p9. Top1 peptide binding was predominantly contributed by HLA-DRs’ nonpolar interactions, which were included into the total binding energy fingerprint of the interface. By investigation of the core nonameric residues of Top1 binding to five different HLA-DRs, the results showed the tendency of p6-p9 binding reduction for HLA-DRB1*01:01 complex based on polymorphic residues at 13, 30 and 74 within pockets. Especially, diversification of hydrophilic residues (C30_*β*_ and S37_*β*_) for the HLA-DRB1*01:01 p9-pocket leads to attract water accessibility across protein-protein interface with a consequence of the interruption of Top1-p9 binding. Moreover, the strong hydrogen bond formation between proteins was mostly found in cases of associated and suspected ATASSc. HLA-DRB5*01:02 was reported to have binding strength for Top1 peptide as strong as susceptible ATASSc group indicating that there should be an appropriate consideration to include this allele into genetic risk for ATASSc development. In our point of views, this information allows us to summarize that HLA-DRs susceptible with ATASSc presents the strong binding affinity for Top1, which well explains the direct relationship between HLA/peptide specific recognition and pathogenicity of SSc disease in a similar manner with the clinically observed information. One of the autoimmunity theories mentioned that the high conservative epitopes between foreign-antigen and self-peptide might be involved in the effective autoimmune disease^38–40^. Identification of foreign-antigen as well as this Top1 self-peptide sequence is necessary to avoid triggering of ATASSc from pathogenic environment, especially for those who had HLA-DR genetic risk for the SSc disease with ATA.

## Materials and Methods

### HLA-DRs/Top1 complex preparation

The 3D structures of the HLA-DRs/Top1 in this study are not available. Amino acid sequences of HLA-DRB1*08:02^41^, HLA-DRB1*11:01^42^, HLA-DRB1*11:04^43^ and HLA-DRB5*01:02^44^ proteins were obtained from the National Center for Biotechnology Information (NCBI) database (http://www.ncbi.nlm.nih.gov/). With over 85% identity, the derived crystal structure from the Protein Data Bank (PDB; http://www.rcsb.org/pdb/), HLA-DR3 carrying CLIP 87–101 peptide (pdb code: 1A6A^45^) was used as the template for HLA-DRB1*08:02, HLA-DRB1*11:01 and HLA-DRB1*11:04, whereas HLA-DRB5*01:01 binding with myelin 86–105 peptide (pdb code: 1FV1^46^) was chosen to construct HLA-DRB5*01:02. Few amino acids in HLA-DR templates were virtually mutated to four HLA-DR/Top1 complexes in this work using the Discovery studio 2.5 (Accelrys, Inc.) executed by the align sequence module. The PDB entry 1AQD^47^ for HLA-DRB1*01:01 carrying an endogenous peptide with 15 amino acids in length was established by changing the original peptide to Top1. Binding between residues of HLA-DR and Top1 spanning RIANFKIEPPGLFRGRGNHP (349–368) sequence is determined by conservation of key anchor sites (p1, p4, p6 and p9)^48^. HLA-DRs in their free form were obtained by removing the antigenic peptide from the binding groove. Both groups of HLA-DR structures, with and without Top1 peptide, were modeled for MD simulations.

### Molecular dynamics (MD) simulation

Assisted Model Building with Energy Refinement (AMBER) version 14 with LEaP module^49^ was employed to add all missing atoms within the starting structures based on the ff03.r1 force field. This version contains the charge set for –COO^−^ and –NH_3_^+^ terminal groups of proteins updated from original ff03 for more accuracy^50^. Added hydrogen atoms were minimized, while the others were frozen. H++ web-prediction of protonation (http://biophysics.cs.vt.edu/H++) was applied to the protein complexes kept at pH 7.0 which were then by randomly neutralized by 7, 7, 7, 12 and 4 sodium ions for HLA-DRB1*08:02, HLA-DRB1*11:01, HLA-DRB1*11:04, HLA-DRB5*01:02 and HLA-DRB1*01:01; respectively. HLA-DRs with and without Top1 peptide were subsequently solvated by TIP3P^51^ water molecules in an orthogonal cage of 97.87 × 109.36 × 78.92 Å^3^. The isothermal-isobaric (NPT) ensemble with constrained number of atoms (N), pressure (P) and temperature (T) was applied in a periodic boundary. The SANDER module of AMBER 14 was used to minimize all water molecules and protein complexes, respectively. The systems were heated to 298 K for 100 ps under constrained atoms of HLA-DR/Top1 by a weak force of 60.0 kcal∙mol^−1^∙Å^2^. The SHAKE algorithm constrained all bonds and angles involving hydrogen atoms with a time step of 2 fs^52^. Long range electrostatic interactions within a cutoff radius of 12 Å were calculated using the particle mesh Ewald (PME) method^53^, while short range non-bonded interactions were evaluated with 12 Å atom-based cutoff for Lennard-Jones potential^51^. MD simulation using the Verlet algorithm^54^ was performed for all HLA-DR/Top1 complexes and the snapshots were stored every 0.2 ps during the operation of 100 ns. The systems of HLA-DR free form have been setup for MD procedure analogously to their complexed structures. The pmemd.cuda module of AMBER was performed in all simulations. In addition, each protein complexes was performed for 10 independent simulations with randomized initial atomic velocities. For analysis, the MD trajectories in production phase were collected for analysis of complex stability, binding free energy, hydrogen bonding and water distribution.

### Binding free energy calculations

Molecular Mechanics/Poisson-Boltzmann Surface Area (MM-PBSA) and Molecular Mechanics/Generalized Born Surface Area (MM-GBSA) methods have been widely employed for calculation of binding free energies of ligand-protein and protein-protein complexes using molecular mechanics (force fields) and implicit solvation models^55–58^. Here, both methods were applied on HLA-DR/Top1 complexes. Average binding free energy (∆*G*_*bind*_) is calculated for each species in the complexed form (*G*_*HLA-DR/Top1*_) and isolated forms consisting of HLA-DRB (*G*_*HLA-DR*_) and Top1 peptide (*G*_*Top1*_) based on the equation below:

1 $$\langle {\rm{\Delta }}{G}_{bind}\rangle =\langle {G}_{HLA-DR/Top1}\rangle -(\langle {G}_{HLA-DR}\rangle +\langle {G}_{Top1}\rangle )$$

Prediction of free binding energy (*G*) is computed by enthalpy (*H*) and entropic (TΔ*S*) terms, according to the principle equation:

2 $$G=H-T{\rm{\Delta }}S$$

Total *H* sums up energy of molecular mechanics (*E*_*MM*_) and free energy of solvation (*G*_*sol*_).

3 $$G=({E}_{MM}+{G}_{sol})-T{\rm{\Delta }}S$$

Where *E*_*MM*_ is divided into van der Waals (*E*_*vdW*_) and electrostatic (*E*_*ele*_) interactions in a couple of complex, as well as internal energy (*E*_*int*_). The execution uses the SANDER module of AMBER14 in gas phase.

4 $${E}_{MM}={E}_{vdW}+{E}_{ele}+{E}_{{int}}$$

5 $${G}_{sol}={G}_{PB(GB)}+{G}_{SASA}$$

Free energy of solvation (*G*_*sol*_) cooperates polar and nonpolar terms from protein in vacuum to solvent transference. Electrostatic contribution can be calculated using PB or GB model. PB model is solved by Poisson-Boltzmann equation to determine molecular charge distribution to the solvation free energy. An alternative method is polarization energy of GB model which is implemented for PB model analytic approximation. The solvent accessible surface area is presented *G*_*SASA*_ to consider proportionate cavity and solute–solvent van der Waals terms using a solvate probe radius of 1.4 Å. The degree of disorder in the systems is examined by the entropic term consisting of rotational, translational and vibrational modes. A part of entropy is driven through expensive normal mode (NMODE) calculation. The normal mode analysis at 298 K was calculated using mmpbsa_py_nabnmode program to derive the entropic term.

Likewise, binding free energy with quantum mechanics (QM) method is computed within the QM/MM-GBSA approach^59,60^. QM/MM approach in a couple with GB model is examined to calculate enthalpy and solvation, respectively. QM calculation based on self-consistent charge density functional tight binding (SCC-DFTB) method^61^ is specifically applied on the hot-spot residues. The semi-empirical implementation of SCC-DFTB is a high level and accurate potential method that can be applied for the calculation of the binding free energy.

## Supplementary information

supplementary info
