# Supplementary material for: Interactions of HLA-DR and Topoisomerase I Epitope Modulated Genetic Risk for Systemic Sclerosis
Source: Sci Rep. 2019 Jan 24;9:745. doi: 10.1038/s41598-018-37038-z (PMC6345791; doi:10.1038/s41598-018-37038-z)
Supplement: Supplementary file 1 — supplementary info [file 41598_2018_37038_MOESM1_ESM.docx]

Supplementary Material

# Interactions of HLA-DR and Topoisomerase I Epitope Modulated Genetic Risk for Systemic Sclerosis

**Sirilak Kongkaew^1,2^, Thanyada Rungrotmongkol^3,4,*^, Chutintorn Punwong^5^**

**Hiroshi Noguchi^6,7^, Fujio Takeuchi^7,8^, Nawee Kungwan^9,10^, Peter Wolschann^2,11,12^,**

**Supot Hannongbua^2,*^**

^1^Program in Biotechnology, Faculty of Science, Chulalongkorn University, Bangkok, 10330, Thailand.

^2^The Center of Excellence in Computational Chemistry, Department of Chemistry, Faculty of Science, Chulalongkorn University, Bangkok, 10330, Thailand.

^3^Biocatalyst and Environmental Biotechnology Research unit, Department of Biochemistry, Faculty of Science, Chulalongkorn University, Bangkok, 10330, Thailand.

^4^Ph.D. Program in Bioinformatics and Computational Biology, Faculty of Science, Chulalongkorn University, Bangkok, 10330, Thailand.

^5^Department of Physics, Faculty of Science, Prince of Songkla University, Hat Yai, Songkhla, 90110, Thailand.

^6^School of Pharmacy, Nihon Pharmaceutical University, Saitama, 361-0806, Japan.

^7^School of Pharmaceutical Sciences, University of Shizuoka, Shizuoka, 422-8526, Japan.

^8^Faculty of Health and Nutrition, Tokyo Seiei University, Tokyo, 124-8530, Japan.

^9^Department of Chemistry, Faculty of Science, Chiang Mai University, Chiang Mai, 50200, Thailand.

^10^Center of Excellence in Materials Science and Technology, Chiang Mai University, Chiang Mai, 50200, Thailand.

^11^Department of Pharmaceutical Chemistry, University of Vienna, Vienna, 1090, Austria.

^12^Institute of Theoretical Chemistry, University of Vienna, Vienna, 1090, Austria.

*Corresponding author

E-mail: (S.H.) [Supot.h@chula.ac.th](mailto:Supot.h@chula.ac.th); (T.R.) t.rungrotmongkol@gmail.com

**Table S1.** HLA-DR sequences are constructed from identical protein templates and used for MD calculation.

| **HLA** | **X-ray structure**  **(PDB code)** | **X-ray template**  **(PDB code)** | **Sequence (Accession no.)** | **% iden. from BLAST search** |
| --- | --- | --- | --- | --- |
| **DRB1*08:02** | - | 1A6A | Q30134 | 85 |
| **DRB1*11:01** | - | 1A6A | CAM84026 | 86 |
| **DRB1*11:04** | - | 1A6A | CAJ01187 | 89 |
| **DRB5*01:02** | - | 1FV1 | BAO73173 | 97 |
| **DRB1*01:01** | 1AQD | - | - | - |

**Table S2.** Sequence alignments among five HLA-DRs are reported as the percentages of identity and similarity.

| **% iden.**  **% sim.** | **DRB1*08:02** | **DRB1*11:01** | **DRB1*11:04** | **DRB5*01:02** | **DRB1*01:01** |
| --- | --- | --- | --- | --- | --- |
| **DRB1*08:02** | - | 97.3 | 96.8 | 89.3 | 90.9 |
| **DRB1*11:01** | 98.9 | - | 99.5 | 89.3 | 90.9 |
| **DRB1*11:04** | 98.4 | 99.5 | - | 88.8 | 90.4 |
| **DRB5*01:02** | 95.7 | 95.7 | 95.2 | - | 92.0 |
| **DRB1*01:01** | 95.7 | 95.7 | 95.2 | 97.9 | - |


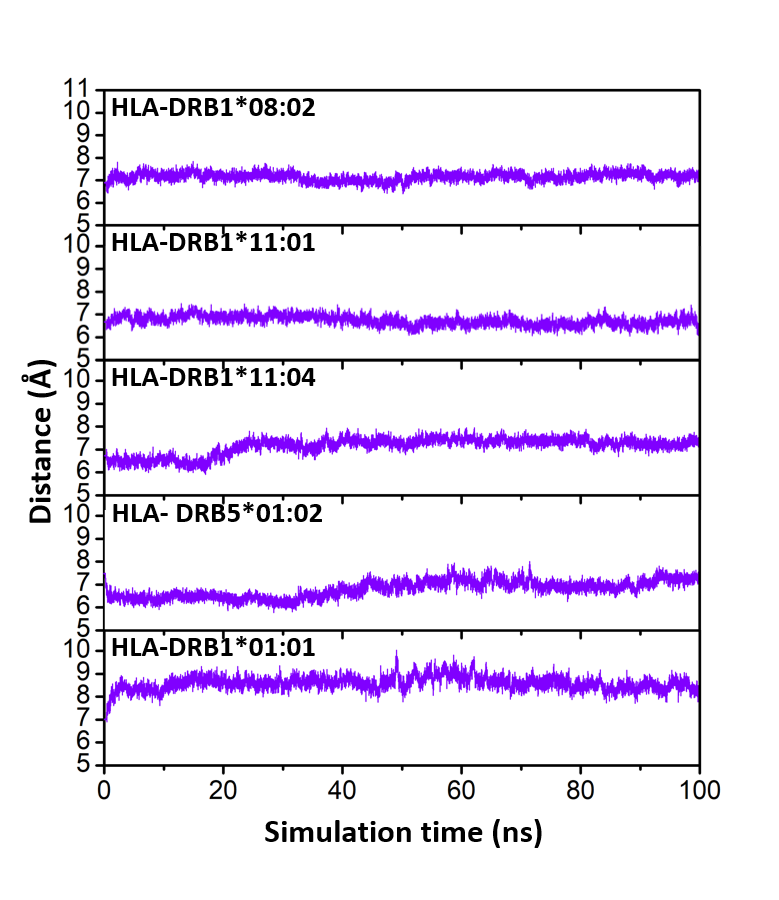


**Figure S1.** **The complex stability are measured as the distances between the centers of gravity of 9-mer core Top1 peptide and HLA binding cleft.**

Distances between the centers of gravity of 9-mer core Top1 peptide and HLA binding cleft are plotted along 100-ns (Fig. S1). The susceptible and suspect HLA-DRs bind Top1 at the distances 6-7 Å closer than the insusceptible HLA-DRB1*01:01 (8-9 Å). The peptide is firmly bound on HLA-DRB1*08:02, HLA-DRB1*11:01 and HLA-DRB1*01:01 binding cleft after 5-ns. HLA-DRB1*11:04/Top1 and HLA-DRB5*01:02/Top1 complex represent more constant distance during 50-100 ns. The distance flexibilities are within ⁓1 Å. After 50 ns, all complexes are in better stability.


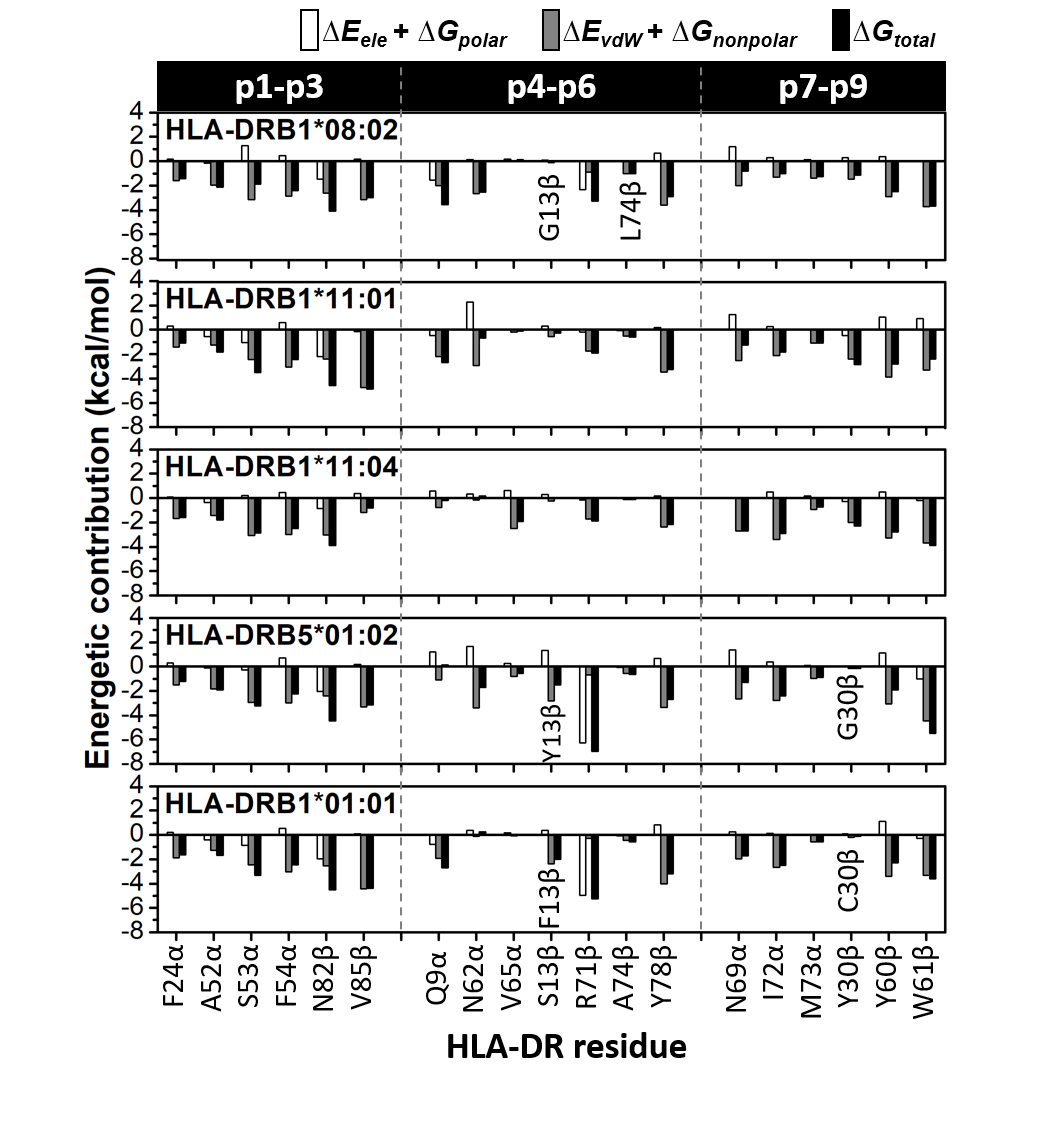


**Figure S2.** **HLA-DR residues interacting with p1 to p9 of Top1 peptide.** Total decomposition energy per-residue (black bar) with contributed by polar (white bar) and nonpolar (gray bar) interactions.


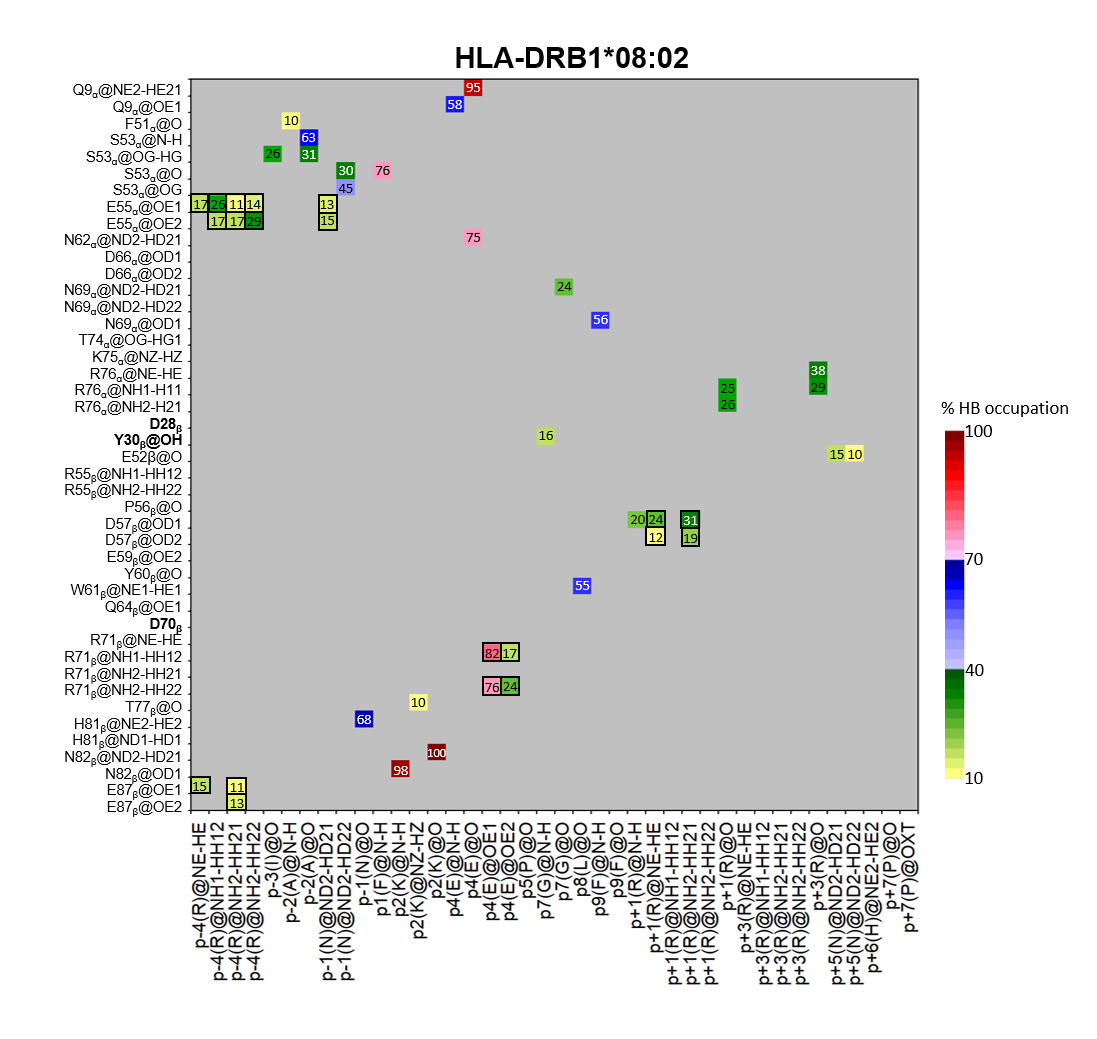


**Figure S3. Overall hydrogen bonding between the whole Top1 peptide and five HLA-DRs.** Polymorphic HLA-DR residues are remarked as the bold text, and salt bridge (SB) interaction is framed by a black border.

**
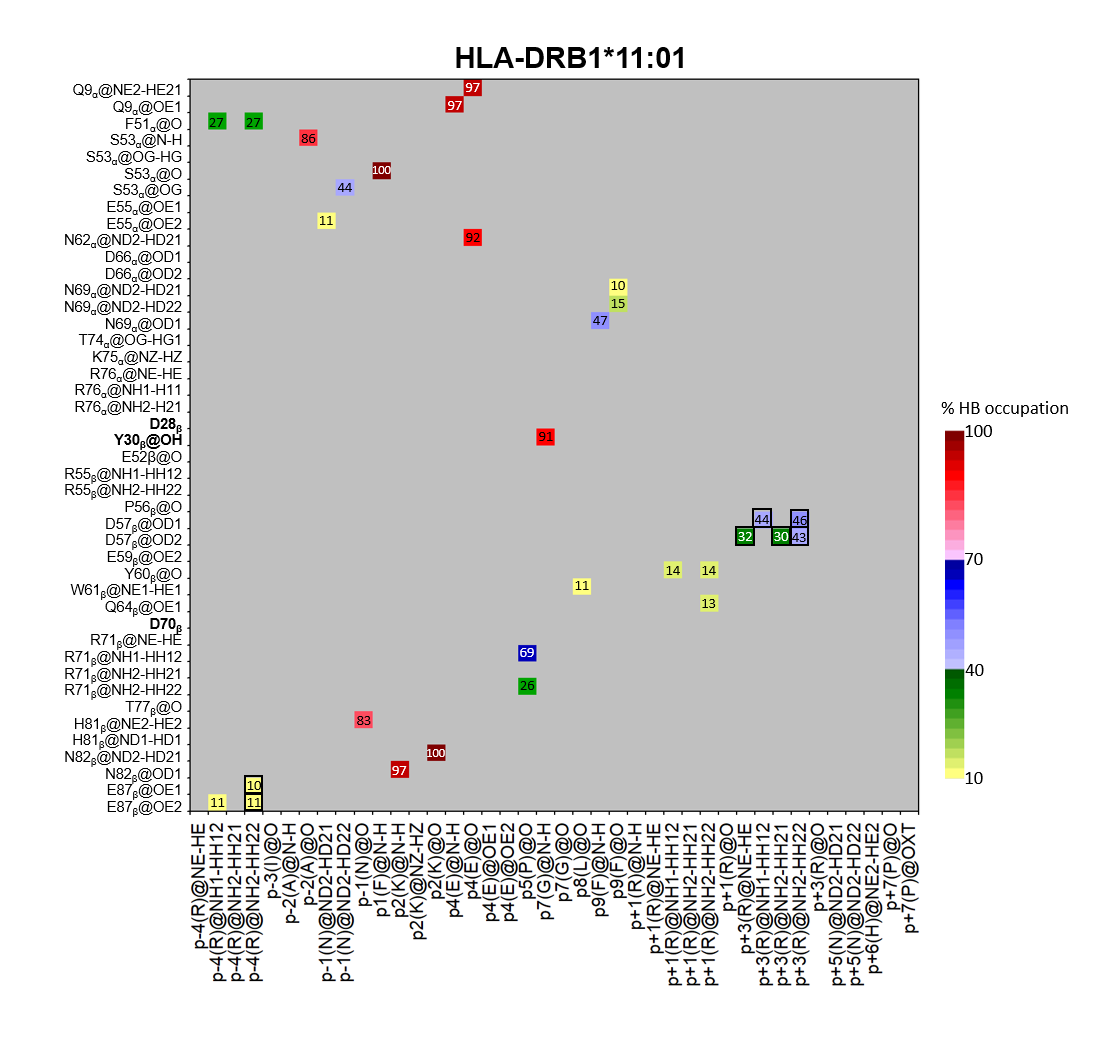
**

**Figure S3.** Cont.


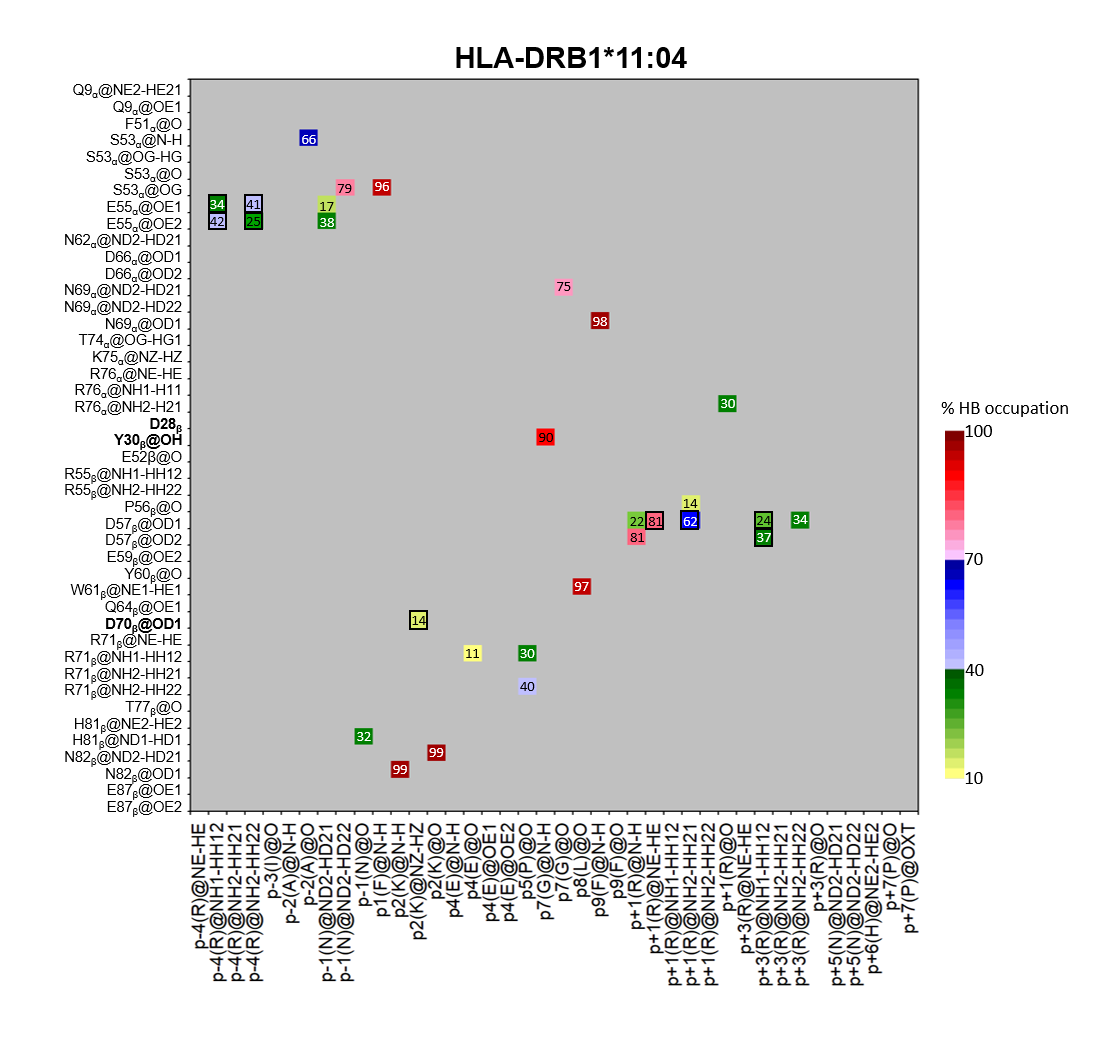


**Figure S3.** Cont.


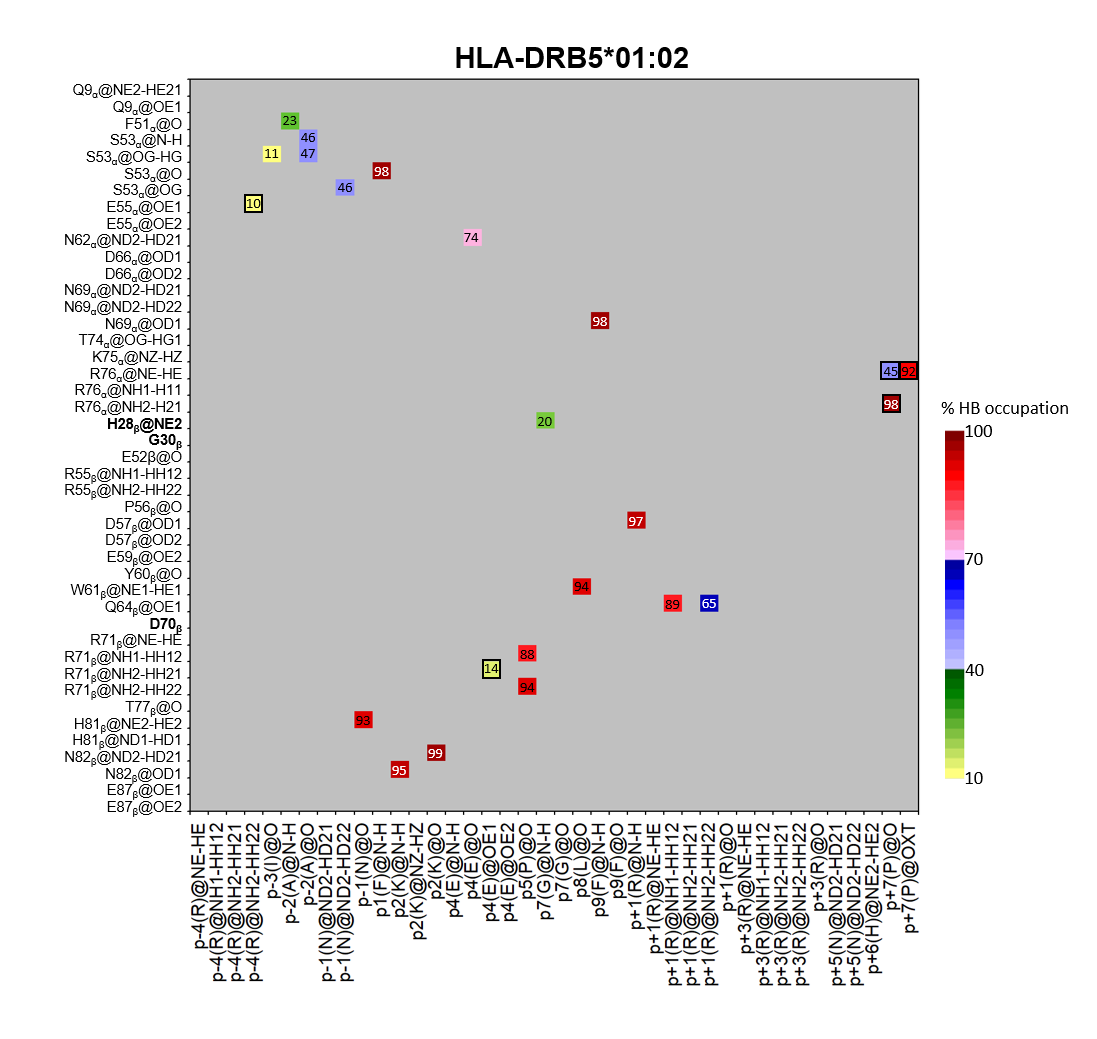


**Figure S3.** Cont.

**
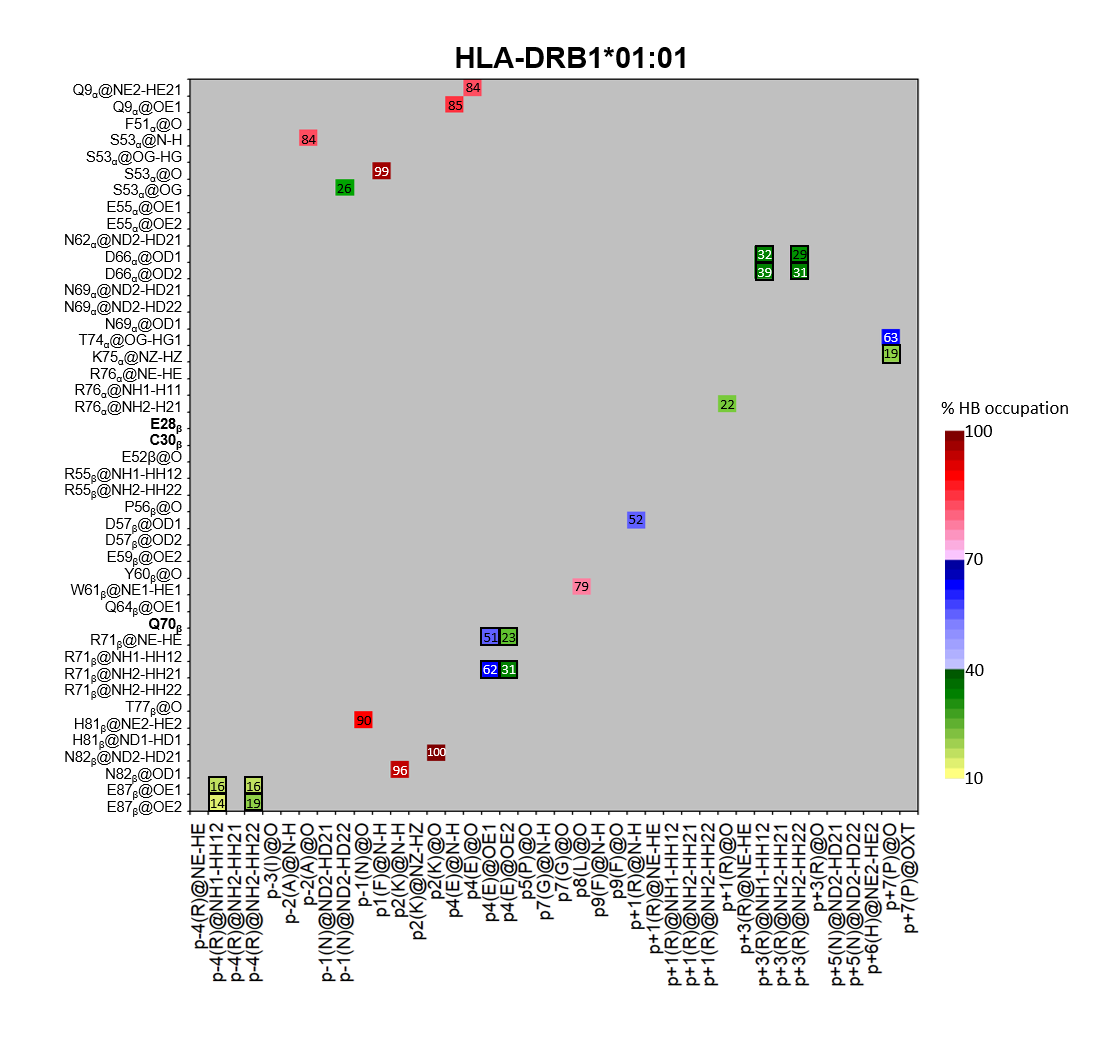
**

**Figure S3.** Cont.
